# From the Infratentorial to the Supratentorial Compartment with a Minimally Invasive Exoscope-Guided Suboccipital Craniectomy: A Case Report and Technical Notes

**DOI:** 10.3390/brainsci15101079

**Published:** 2025-10-05

**Authors:** Giada Garufi, Alfredo Conti, Domenicantonio Collufio, Domenico Matalone, Antonio Morabito, Francesco Messineo, Giuseppe Ricciardo, Salvatore Cardali

**Affiliations:** 1 Department of Neurosurgery, Azienda Ospedaliera Papardo, University of Messina, 98158 Messina, Italyscardali@unime.it (S.C.); 2 Department of Neurosurgery, IRCCS Istituto delle Scienze Neurologiche di Bologna, 40139 Bologna, Italy; 3 Dipartimento di Scienze Biomediche e Neuromotorie (DIBINEM), Alma Mater Studiorum Università di Bologna, Via Altura 3, 40123 Bologna, Italy; 4 Department of Biomedical, Dental and Morphological and Functional Imaging, University of Messina, Via Consolare Valeria, 98125 Messina, Italy

**Keywords:** exoscope, brain tumor, neuroncology, technology in neurosurgery

## Abstract

Background: Surgical exoscopes represent a significant advancement in neurosurgical procedures, offering enhanced visualization through 3D high-definition digital imaging and superior ergonomics. While their adoption is increasing, the full scope of applications and advantages evident in posterior fossa prone suboccipital approaches remains limited. Case Description: We present a detailed technical report of a minimally invasive exoscope-guided suboccipital approach for the resection of a large tentorial meningioma extending into both supra- and infratentorial compartments. Results: The exoscope’s long working distance and co-axial illumination supported circumferential dissection of the tumor–arachnoid interface with reduced instrument–optics conflict and fewer scope repositioning events. Team visualization via shared display improved coordination during hemostasis and capsule mobilization. The early outcome was favorable; the 18-month MRI (added) showed no residual or recurrence. Conclusions: In a prone suboccipital approach, the exoscope enabled stable depth cues in a deep, narrow corridor, minimized optics interference, and enhanced team coordination. These case-specific findings clarify how exoscope features can translate to operative efficiency in posterior fossa surgery while underscoring the need for prospective, comparative, and cost-effectiveness studies.

## 1. Introduction

The surgical management of complex intracranial lesions spanning multiple compartments presents unique challenges that demand both technical expertise and optimal visualization. Recent advances in surgical technology, particularly the development of high-definition 3D exoscopes, have revolutionized the approach to such cases. These systems offer several advantages over traditional microscopes, including improved ergonomics, superior depth perception, and enhanced teaching capabilities.

Managing complex intracranial lesions that span infratentorial and supratentorial compartments poses unique challenges related to exposure, working angles, and operative endurance.

Over recent decades, skull base surgery has evolved through advances in optics and approaches—from operative microscopes and classic microsurgical corridors to expanded endoscopic and exoscopic visualization that decouple eye-to-ocular constraints. Parallel shifts are documented in other skull base pathologies, such as trigeminal schwannomas [1], where classic microsurgical approaches were benchmarked in large series and, more recently, endoscopic and endoscope-assisted techniques helped reshape surgical paradigms by improving access/angles and team visualization. These developments contextualize exoscope adoption by highlighting how visualization platforms can redefine ergonomics, corridor selection, and educational value in complex skull base workflows.

Digital exoscopes have emerged as a useful evolution of the operating microscope, delivering high-definition, three-dimensional (3D) visualization with extended depth of field, robust illumination, and a wider working distance. These systems enable heads-up surgery with an externally shared display, improving surgeon posture, reducing cervical strain during prolonged procedures, and enhancing intraoperative teaching and team situational awareness. Contemporary platforms—such as exoscopes—integrate fluorescence capabilities, including 5-aminolevulinic acid guidance, supporting tumor visualization and resection.

In cranial base and posterior fossa surgery, the exoscope’s maneuverability allows rapid camera repositioning without altering the surgeon’s body position, preserving an unimpeded view in deep, narrow corridors. When the visual axis is horizontal, operators can maintain a comfortable frontal posture, often using shorter instruments and relying on gravity-assisted cerebellar relaxation rather than fixed retraction. These workflow characteristics can translate into stable microsurgical technique and reduced operator fatigue while maintaining patient safety.

Tentorial meningiomas that extend across the tentorial incisura have traditionally required extensive craniotomies to secure multidirectional access. Advances in visualization and microsurgical strategies have renewed interest in focused, minimally invasive routes that preserve surgical control while limiting approach-related morbidity. In this report, we present a 60-year-old woman with a large tentorial meningioma measuring 50 × 44 × 43 mm with both infratentorial and supratentorial components. We detail an exoscopic median suboccipital approach (MSA) to the posterior cranial fossa with supratentorial reach, highlighting the technical nuances, ergonomic advantages, and practical limitations of the exoscope in this setting. We also discuss how these features relate to the current literature on exoscopic neurosurgery and skull base pathology.

## 2. Case Summary

A 60-year-old female was admitted to our institution for an increasing history of headache, nausea, and dizziness, which she stated had been present for at least 1 year. There was no complaint of stability issues, no history of trauma, and the patient was otherwise in good health.

### 2.1. Preoperative Considerations

Preoperative evaluation included neurological examination, cranial nerve assessment, and MRI with contrast (axial, coronal, sagittal T1 post-contrast; T2/FLAIR to assess edema; MRV if venous sinus proximity; CTA/MRA as indicated).

During the neurological physical examination, the patient demonstrated a positive Romberg test, ataxia, right dysmetria and adiadochokinesia, speech disorders, and segmental weakness.

**Figure 1:**
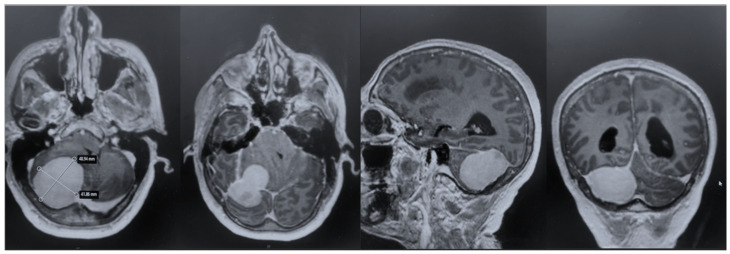
Preoperative MRI + C: a posterior fossa extra-axial mass was demonstrated, showing a low SI on T1 and intermediate SI on T2, which avidly enhanced post-Gad administration. The mass was broad-based on the right tentorium cerebelli with a discrete supratentorial extension, thus compressing the adjacent right cerebellar hemisphere, the brainstem, the IV ventricle, and the right occipital lobe.

After admission, the patient underwent a cerebral MRI, which showed a huge extra-axial lesion in the posterior cranial fossa (5 × 4.4 × 4.3 cm). The lesion demonstrated low SI on T1 and intermediate SI on T2 and avidly enhancing post-Gad administration. The mass showed no restriction on DWI. The mass was broad-based on the right tentorium cerebelli with a discrete supratentorial extension, compressing the adjacent right cerebellar hemisphere, the cerebellar peduncle, the brainstem, the IV ventricle, and the right occipital lobe. A characteristic dural tail sign was demonstrated on post-Gad sequences (Figure 1).

### 2.2. Procedure Selection

For this tentorial meningioma with both supra- and infratentorial extension, we selected an exoscope-guided approach based on (i) an anticipated deep, narrow working corridor; (ii) the need for circumferential arachnoid plane dissection in multiple working angles; (iii) the benefit of shared visualization for assistant and scrub nurse; and (iv) surgeon ergonomics for prolonged dissection.

### 2.3. Anesthesia

Total intravenous anesthesia (TIVA) was selected in order to reduce brain swelling and optimize neuromonitoring. Controlled ventilation targeted normocapnia to mild hypocapnia. Prophylactic antiemetics and steroid protocols were as per institutional practice; antibiotic prophylaxis was administered within 60 min pre-incision.

### 2.4. Precautionary Measures

An external ventricular drainage (EVD) positioning was considered for anticipated tight posterior fossa or hydrocephalus risk; the criteria included ventriculomegaly on MRI or anticipated brain swelling. Neuronavigation was registered on the preoperative MRI. Intraoperative neuromonitoring (IONM), including brainstem auditory evoked potentials (BAEPs) and facial/lower cranial nerve EMG, was performed in order to prevent intra- and postoperative complications. Stepwise bipolar coagulation, hemostatic agents (oxidized cellulose, fibrin sealant) at venous oozing sites, avoidance of overcoagulation near tentorial leaflets, and warm irrigation to maintain a clear field were also adopted as pre- and intraoperative precautionary measures.

### 2.5. Surgical Procedure and Application of the Exoscope

**Figure 2:**
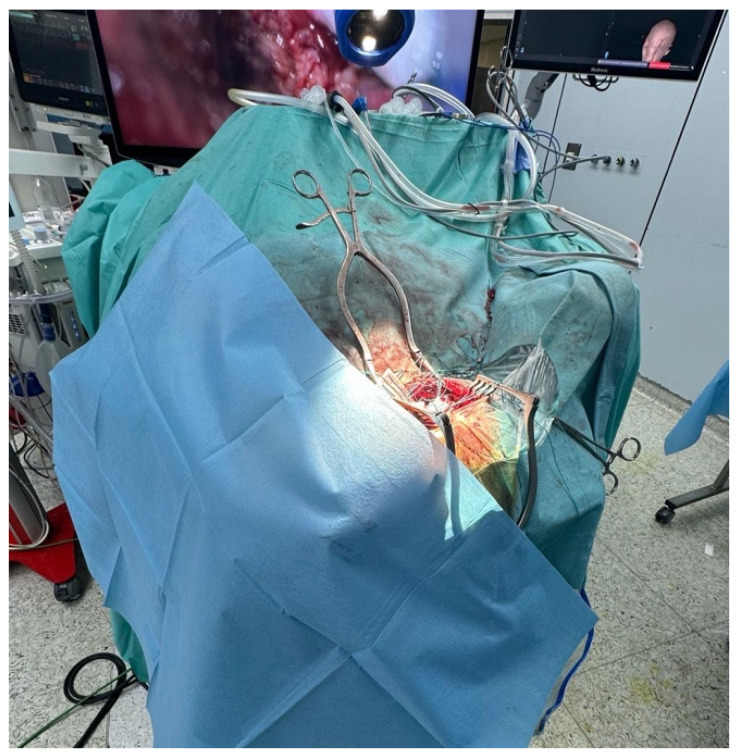
The patient was positioned prone with the head secured in a three-pin clamp, neck gently flexed to elevate the suboccipital region while preserving venous return and avoiding jugular compression. A midline incision from above the inion toward C2 allowed subperiosteal exposure of the occipital squama and, as needed, the posterior arch of C1. A median suboccipital craniectomy was fashioned to the margins of the transverse–sigmoid sinuses, with meticulous hemostasis. The dura was opened in a Y-shaped fashion based on the transverse sinus, and stay sutures elevated the leaflets to widen the corridor. Controlled CSF release from the cisterna magna achieved cerebellar relaxation, minimizing the need for fixed retraction. The exoscope was positioned at a long working distance with co-axial illumination to maintain depth cues and reduce instrument–optics conflict during exposure. Neuronavigation and neuromonitoring were used for orientation and cranial nerve safety during the opening phase.

After general anesthesia, the patient underwent surgery for the exeresis of the lesion. The patient underwent an EVD drainage positioning in the frontal horn of the right lateral ventricle. Then, the patient was positioned prone with the head secured in a three-pin holder, neutral to slight flexion to align the suboccipital corridor and minimize venous congestion (Figure 2).

**Figure 3:**
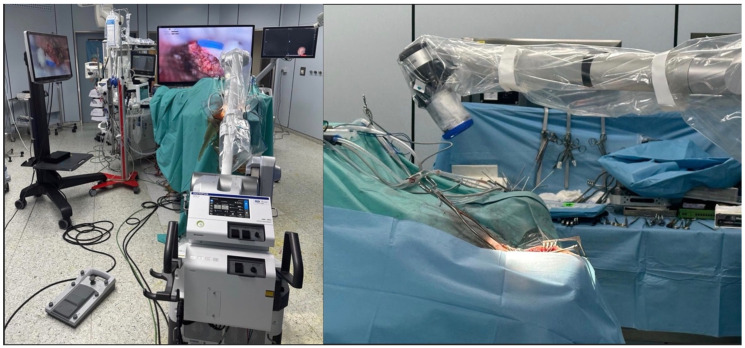
Intraoperative setting. The body of the exoscope was placed behind the operative field. Then, guiding the arm of ORBEYE beyond the patient’s body, the scope was placed around the operative field at a distance of about 15–20 cm; the monitor was placed beyond the patient, facing the surgeon. The surgeons, the nurses, and the residents watched the surgery, thanks to the frontal 3D 4k screen. The use of the exoscope guaranteed proper ergonomics for the surgeons during the entire procedure, a 360° view of the lesion, thanks to the telescopic camera, and a clear view of the supratentorial portion, thanks to the 30–45° angulation of the camera.

The exeresis was performed with an exoscope-guided approach (Olympus Orbeye, Olympus Inc., Tokyo, Japan) in order to achieve the radical excision of both the infratentorial and the supratentorial portion of the lesion. The body of the exoscope was placed behind the operative field. Then, guiding the arm of ORBEYE beyond the patient’s body, the scope was placed around the operative field at a distance of about 15–20 cm; the monitor was placed beyond the patient, facing the surgeon. The surgeons, the nurses, and the residents watched the surgery thanks to the frontal 3D 4k screen. The use of the exoscope guaranteed proper ergonomics for the surgeons during the entire procedure, a 360° view of the lesion, thanks to the telescopic camera, and a clear view of the supratentorial portion, thanks to the 30–45° angulation of the camera (Figure 3). Using ORBEYE, even when the angle of the operative visual axis was approximately horizontal, the surgeons were able to perform manipulations in an ergonomic posture; they were not forced to work in an uncomfortable posture, avoiding extension of the arms, as often happens with a conventional microscope.

The procedure was completed in 8 h.

### 2.6. Postoperative Course

The patient underwent ICU observation for the first 12 h with frequent neurological checks, a head elevation of 30 degrees, normotension, analgesia, and antiemesis. Then, the patient was moved to the clinical ward with early mobilization to avoid thrombosis events.

**Figure 4:**
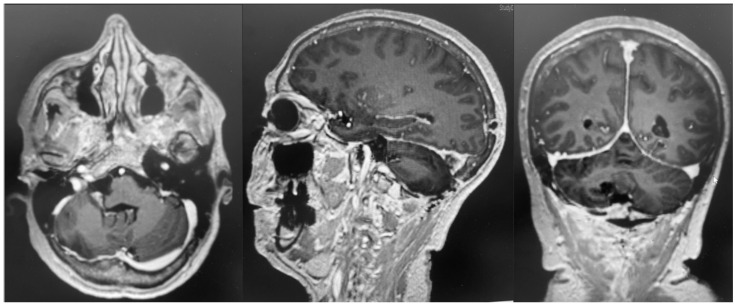
Postoperative MRI + c: these pictures show the gross total resection of the lesion with no signs of postoperative complications.

The patient underwent an early postoperative MRI, which documented the complete resection of the lesion without any postoperative complications (Figure 4). The patient was discharged with a significant improvement in the neurological examination at 7 days after surgery. Histopathology confirmed a diagnosis of meningioma, WHO grade 1, subtype fibrous.

**Figure 5:**
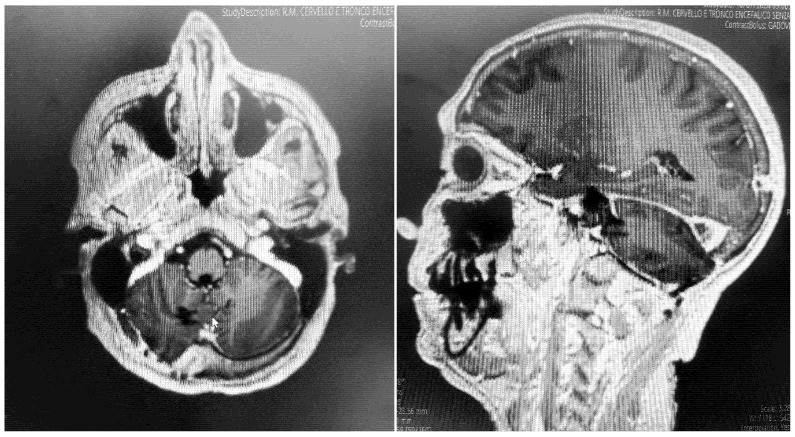
Post-contrast T1-weighted MRI at 18 months following resection. Axial and sagittal images at the level of the prior tumor demonstrate no nodular or mass-like enhancement to suggest residual or recurrent meningioma.

To strengthen the oncologic perspective, we added an 18-month contrast-enhanced MRI, demonstrating no residual or enhancing recurrence (Figure 5). While early postoperative findings were favorable, meningiomas require longer-term surveillance. Our current imaging aligns with standard practice and supports durable tumor control at 1 year. Continued surveillance will follow institutional protocol, with annual MRI for at least 5 years, tailored to WHO grade, Simpson resection grade, and histopathology.

## 3. Discussion

This is the first study, to our knowledge, to report the feasibility of a suboccipital approach in a prone position using the Orbeye exoscope in a tentorial meningioma extended to the supratentorial region.

Exoscopes have emerged as digital visualization platforms offering high-definition, three-dimensional (3D) or two-dimensional (2D) imaging, extended depth of field, and improved ergonomics compared with conventional operating microscopes [2,3,4]. In complex skull base and posterior fossa surgery, where narrow corridors, steep working angles, and prolonged operative times are common, exoscopes may enhance visualization and surgeon comfort while maintaining surgical precision [3,5]. The present case illustrates these advantages in the resection of a tentorial meningioma spanning infratentorial and supratentorial compartments via a minimally invasive median suboccipital approach.

Multiple comparative series report that modern 3D exoscopes provide image quality comparable to operating microscopes, with superior illumination, magnification at deep fields, and wider working distances [2,4,6]. Surgeons frequently report reduced cervical strain and improved posture due to heads-up display, which may mitigate fatigue during lengthy skull base procedures [3,7]. Depth perception with 3D systems is generally rated favorably after a brief acclimatization period, although 2D exoscopes may limit stereopsis. For deep-seated lesions and steep angles around the tentorial incisura, the ability to reposition the camera without moving the surgeon’s body is repeatedly cited as beneficial [5,8].

Prospective and retrospective cohorts in cranial and spinal procedures suggest noninferior operative times, blood loss, and complication rates when using exoscopes versus microscopes [2,3,4,9]. Learning curves are evident but typically short; surgeons gain efficiency after several cases [7,9]. Case series in meningioma, vestibular schwannoma, aneurysm clipping, and bypass report satisfactory extent of resection or clip placement with preserved neurovascular safety [6,8,10]. For posterior fossa meningiomas, exoscopic approaches have enabled comfortable resection under high magnification with consistent identification of neurovascular structures and arachnoid planes [5,6].

Because of the outer exoscope display, assistants and trainees view the same field in real time, improving anticipation of surgical steps and facilitating teaching [7,11]. This shared view can streamline instrument passing and suction/irrigation coordination. However, operating room layout and monitor placement require preoperative planning to avoid line-of-sight conflicts. Some reports note increased instrument collisions early in adoption, which diminish with experience [6]. The reported limitations include occasional glare or washout on highly reflective surfaces, dependence on display resolution and brightness settings, and reduced haptic cues due to camera distance [3,4]. Integration with neuronavigation and fluorescence (e.g., indocyanine green, 5-ALA) is increasingly available but not universal across platforms [8,10]. Cost and the need for staff training are nontrivial. Exoscope platforms typically require a substantial capital investment (often mid to high six figures, depending on the configuration), along with recurring expenses (service contracts, disposables, staff training, and potential integration costs with visualization/recording infrastructure). Justifying adoption, therefore, hinges on demonstrable value: operative efficiency (reduced repositioning and setup time), surgical ergonomics (mitigation of musculoskeletal strain), enhanced team visualization and training, high-quality documentation/tele-mentoring, and potentially reduced turnover for multi-specialty shared-use. Institutions may consider shared or hybrid models (exoscope plus existing microscope) while prospective data are gathered. Formal cost-effectiveness analyses across procedure types and case volumes are needed to define where the exoscope delivers net value [9].

For extremely narrow corridors, traditional microscopes or endoscopes may still be preferred. Hybrid strategies that combine an exoscope with an endoscope or a microscope are common in complex skull base surgery [5,6].

Systematic reviews conclude that exoscopes are safe and effective alternatives to operating microscopes with comparable complication rates and extent of resection across cranial and spinal indications [3,4,5]. Randomized data are scarce; most evidence derives from observational studies and learning curve analyses. In skull base pathology, including tentorial and petroclival meningiomas, small case series consistently report favorable visualization, surgeon comfort, and satisfactory oncological and functional outcomes, with no clear signal of increased morbidity [5,6,8,11].

Beyond general advantages, our findings map directly onto observed exoscope performance in deep cranial corridors. First, we noted preserved depth perception and plane fidelity during circumferential dissection of the tumor–arachnoid interface, with fewer optics-related repositioning events than we typically encounter with a microscope in suboccipital exposures. This is concordant with comparative and prospective series reporting comparable or superior visualization and assistant view quality, even in narrow operative fields [7,12,13]. Second, instrument–optics interference was minimal because the camera remained outside the working cone; this echoes reports of reduced instrument collisions and improved access angle flexibility [14]. Third, real-time shared visualization improved assistant task anticipation and teaching, consistent with studies highlighting educational and team benefits of 3D/4K exoscope displays [8,10]. Finally, our workflow benefitted from rapid zoom/focus transitions during hemostasis and capsule mobilization—steps in which prior studies note that stable magnification and lighting can mitigate the depth-perception learning curve [12,13,14]. Together, these case-specific elements clarify how exoscope features translate to practical gains in a prone suboccipital approach and help reconcile mixed findings on depth cues by emphasizing technique (camera angle, distance, and display calibration) and team adaptation. In this patient with a large tentorial meningioma extending across the tentorial incisura, the exoscope supported safe dissection in deep and angled corridors while maintaining ergonomic posture over a prolonged procedure. High-definition magnification facilitated internal debulking, arachnoid plane preservation, tentorial detachment, and protection of the cerebellum and brainstem. These features align with published reports emphasizing deep-field illumination, ease of camera repositioning, and reduced surgeon fatigue [3,5,6,7].

This single-case report establishes feasibility rather than comparative efficacy or safety. The favorable outcome may reflect tumor- and patient-specific factors (consistency, vascularity, attachment pattern) and surgeon experience. As such, generalizability is limited, and variability across tentorial meningiomas can substantially alter exposure, hemostasis, and resection risk. Future prospective cohorts or registries comparing exoscope- and microscope-assisted resections are needed to assess outcomes, complications, operative time, and learning curve effects.

## 4. Conclusions

This study highlights the feasibility and clinical applicability of the Orbeye exoscope for performing a suboccipital approach in the prone position for tentorial meningiomas with supratentorial extension. The use of the exoscope introduces several practical advantages over traditional operating microscopes, particularly in terms of ergonomic comfort, intraoperative flexibility, and reduced strain on the surgical team. The stereoscopic 360° visualization and the rotatable camera head allow the surgeon to maintain an optimal posture regardless of the visual axis, which is particularly beneficial during long and complex procedures involving deep or angled surgical corridors.

Our experience suggests that, in a prone suboccipital posterior fossa approach, the exoscope supported stable depth perception in a deep corridor, reduced instrument–optics conflict, and improved team coordination, facilitating efficient circumferential arachnoid plane dissection. One-year imaging demonstrated durable radiographic control in this case. These findings suggest exoscope feasibility and workflow advantages in posterior fossa surgery while motivating prospective studies addressing efficiency, ergonomics, and cost-effectiveness.

## Data Availability

The original contributions presented in this study are included in the article. Further inquiries can be directed to the corresponding author.
